# Construction and analysis of cDNA libraries from the antennae of *Batocera horsfieldi* and expression pattern of putative odorant binding proteins

**DOI:** 10.1093/jis/14.1.57

**Published:** 2014-01-01

**Authors:** Hui Li, Aijun Zhang, Li-Zhen Chen, Guoan Zhang, Man-Qun Wang

**Affiliations:** 1 Hubei Insect Resources Utilization and Sustainable Pest Management Key Laboratory, College of Plant Science and Technology, Huazhong Agricultural University, Wuhan 430070, P. R. China; 2 Henan University of Technology, Zhengzhou 450001, P. R. China; 3 Invasive Insect Biocontrol and Behavior Laboratory, USDA-ARS-Plant Sciences Institute, Beltsville, MD 20705- 2350, USA

**Keywords:** Antennal cDNA library, Minus-C OBPs, RT-PCR, qPCR

## Abstract

A high-quality cDNA library was constructed from female and male antenna of the longhorned beetle, *Batocera horsfieldi* (Hope) (Coleoptera: Cerambycidae), a serious pest of *Populus* (Salicales: Salicaceae). The titer was approximately 2.37 × 106 pfu/mL, and this complies with the test requirement. From the libraries, 692 clones were selected randomly, sequenced, and further analyzed, and the recombinational efficiency reached 93.85%. By alignment and cluster analysis, we identified four odorant binding proteins, two pheromone-binding proteins (have the characteristic six conserved cysteine residues), four Minus-C odorant binding proteins (lost two conserved cysteines), and three chemosensory proteins. In this study, we describe the identification and characterization of four new cDNAs that encode Minus-C odorant binding proteins (Minus-C OBPs) from *B. horsfieldi* antennal cDNA libraries. Our investigation focused on the expression pattern of the Minus-C OBP genes in various tissues in both sexes at different developmental stages, using reverse transcription PCR (RT-PCR) and realtime PCR (qPCR) strategies. Minus-C OBP1, 2, and 3 were expressed in all tested tissues, with the exception of the head (without antenna, labial palps, and maxillary palps). Minus-C OBP4 was expressed in the antenna, legs, and abdomen, but not in the labial palps, maxillary palps, or head. The qPCR results revealed MinusC OBPs were expressed in the antenna throughout the adult life, and that the transcript levels of these genes depended on the sex, age, and mating status of adults.

## Introduction

The sense of smell is vital in insect communication, and most insect species’ chemoreception systems are extremely sensitive to environmental odors and tastes. Considerable progress has been made in understanding insect olfaction, and it is known that odorant binding proteins (OBPs) and odorant receptors play important roles in this process ( Zwiebel and Takken 2004 ). There are a large number of OBPs present within a variety of insect species. Insect OBPs are small, globular, water-soluble proteins that are specifically expressed in both the olfactory and gustatory systems ( Vogt and Riddiford 1981 ; Steinbrecht et al. 1996 ). OBPs of insects were first identified in the silkmoth, *Antheraea polyphemus* , where they are known as pheromone binding proteins (PBPs) ( Vogt and Riddiford 1981 ). Subsequently, large families of similar but divergent OBPs have been identified in many other insect species ( Pelosi and Maida 1995 ; Krieger et al. 1997 ; Vogt et al. 1999 ; Hekmat-Scafe et al. 2000 ; Ishida et al. 2002 ; Nagnan-Le Meillour 2004 ; Zhang et al. 2009a , b).

The features identifying genes encoding OBPs include the six-cysteine signature, a size of 15-20 kDa, the α -helix pattern, the globular water-soluble nature, and the presence of a signal peptide. A six-cysteine signature motif is the most typical, and generally diagnostic, feature of the classical insect OBPs ( Pelosi and Maida 1995 ). The spacing pattern of the conserved cysteine residues in Coleoptera is typically C1-X23-44-C2-X3-C3-X36-43-C4-X8-12- C5-X8-C6, in which X is any amino acid ( Xu et al. 2009 ). The OBPs of insect are a multigene family that includes a lot of members: most OBPs are classic OBPs (possessing all the features), seem to play a more general role in olfaction by carrying odorants ( Vogt and Riddiford 1981 ; Vogt and Lerner 1989 ) and perceiving sex pheromones Vogt et al. 1991 ), dimer OBPs (having two six-cysteine signatures), plus-C OBPs (having two additional conserved cysteines plus 1 proline), Minus-C OBPs (having lost two conserved cysteines), and atypical OBPs (having nine to 10 cysteines and a long C-terminus) ( Hekmat-Scafe et al. 2002 ; Xu et al. 2003 ; Zhou et al. 2004 ).

The longhorned beetle, *Batocera horsfieldi* (Hope) (Coleoptera: Cerambycidae), is an important pest of *Populus* (Salicales: Salicaceae) species. The larvae and pupae develop inside *Populus* , and every year in May in China the adults of *B. horsfieldi* eclose, and newly-emerged adults use semiochemicals from *Rosa multiflora* Thunb (Rosales: Rosaceae) to locate their feeding-plant. After mating, the females travel back to *Populus* for oviposition ( Li et al. 2008 ; Zhuge et al. 2010 ). This process allows the development of pest control measures based on olfactory-mediated behavioral modification. Insect antennae contain a high concentration of OBPs, which are believed to be involved in the first step of olfactory molecular recognition and signal transduction by ferrying airborne host odorants across the sensillum lymph to the odorant receptors (ORs) ( Vogt et al. 1981 ; Vogt et al. 1991 ; Pelosi et al. 2006 ). Expressed sequence tags (ESTs) are short, single-pass sequences generated from either the 5' or the 3' end of cDNAs. ESTs of several thousand randomly chosen clones from the cDNA library analyses of certain tissues or organs are useful in identifying a gene or protein system with a specific function, such as chemoreception ( Robertson et al. 1999 ; Zhou et al. 2010 ). In this study, we constructed an antennal cDNA library of *B. horsfieldi* for EST sequencing *.* In order to exploit the molecular mechanism of the perception of volatile cues associated with Minus-C OBP genes, we investigated the expression of Minus-C OBPs in different tissues and both sexes at different development stages in antennae. Phylogenetic relationships of the Minus-C OBPs in *B. horsfieldi* were also analyzed with other OBPs, and the evolution of Minus-C OBP genes was discussed.

## Materials and Methods

**Insect collection and tissue preparation**

*B. horsfieldi* adults were collected from their feeding-plant *R. multiflora* in Gong’an county of Hubei Province in China (112°23′E; 30°04′N). Adult *B. horsfieldi* were determined to be either mated or unmated based on the presence or absence of patch of sexuality. All the insects were individually maintained in clear plastic containers (5 cm in diameter, 8 cm in height) and fed twigs (without leaf) of *R. multiflora* . The antennae from male and female adults were mixed for constructing the cDNA library. For tissue profiling, the antennae, head (without antennae, labial palps, and maxillary palps), labial palps, maxillary palps, mid-abdomen, hind-abdomen, legs (separating foreleg, middle leg, and hind leg), and wings were dissected from the adults of mated males five days after eclosion. Also, for spatial expression, antennae were isolated from the adult virgin males and females and mated males and females at different development stages after eclosion. Tissues were stored at 70oC until used.

### RNA extraction and construction of the antennal cDNA library

For construction of the antennal cDNA library, antennae (1:1 male:female) were quickly ground in liquid nitrogen, then the powder was transferred into a 1.5 mL RNasefree tube and mixed with 1.0 mL Trizol Reagent (Invitrogen, Life Technologies, www.lifetechnologies.com ), following the manufacturer’s instructions. The isolated total RNA from *B. horsfieldi* was reverse transcribed to double-strand cDNA using a modification of the SMART cDNA method (Clontech, www.clontech.com ). A primer containing an oligo (dT) and a unique S *fi* I site at the 3' end was used to prime the first cDNA strand. A second oligonucleotide containing a unique S *fi* I site was added to the 5' cap at the end of the first-strand synthesis. After first- strand cDNA synthesis, long-distance PCR was used for generating the double-strand cDNA. The amplified double-strand cDNA was digested using S *fi* I and size fractionation using CHROMA SPIN Columns (Clontech). In order to construct an antennal cDNA library containing as much of the target genes as possible, 300–1000 bp fragments were collected. Then the cDNA fragments were ligated into the S *fi* I predigested pDNR-LIB (Invitrogen) plasmid vector. The ligation mixture was transformed into competent *DH10* cells and plated on the agar plates supplemented with chloramphenicol (34 µg/mL). The plate was then inverted and incubated at 37°C. The entire step follows the SMART cDNA library construction kit user manual (Clontech).

Total RNA was isolated from each tissue using the Trizol reagent (Invitrogen) and was treated with DNase I (Qiagen, www.qiagen.com ). First-strand cDNA was synthesized using oligo (dT) primer and RevertAid M-MuLV Reverse Transcriptase (Thermo Scientific, www.thermoscientificbio.com ) in a total volume of 20 µL. The mix was incubated at 42°C for 1 hr, and the reaction was terminated by heating at 70°C for 5 min (MBI Fermantas, Thermo Scientific). cDNA was stored at 20°C.

### Clone detection and sequence annotation

The 692 randomly selected clones from the antennal cDNA libraries were amplified by colony PCR using the M13 universal primer. Sequences from the cDNA inserts were determined using ABI 3730 sequencer (Applied Biosystems, Life Technologies). Manually trimming the sequences to remove vector and primer sequences, a total of 402 informative ESTs (unigenes) were obtained. To search for homologous olfactory genes, all nucleotide sequences were subjected to the Basic Local Alignment Search Tool (BLASTx and BLASTn, NCBI, www.ncbi.nlm.nih.gov ). Olfactory genes were identified by their characteristic features, containing six or four conserved cysteines. The signal peptides and cleavage sites were predicted using SignalP 4.0 (Center for Biological Sequence Analysis, Technical University of Denmark, www.cbs.dtu.dk/services/SignalP-4.0 ; Nielsen et al., 1997 ). In order to compare sequence similarity among homologous genes, Minus- OBP-related sequences from *Tribolium castaneum* (Herbst) (Coleoptera: Tenebrionidae) and other species were initially identified using the NCBI BLAST network server and retrieved from GenBank (NCBI). All sequences were aligned using ClustalX ( www.clustal.org ; Thompson et al. 1994 ). The neighbor-joining method was used to construct the phylogenetic tree using MEGA version 4.0 ( www.megasoftware.net ; Tamura et al. 2007 ). Bootstrapping was performed to estimate the reliability of the branches using 1500 neighbor-joining replicates. The tree presented only includes nodes with 50% or higher bootstrap support.

### Tissue specificity and spatial expression profiling of *B. horsfieldi* Minus-C OBPs

To investigate tissue and spatial expression profiling of Minus-C OBPs, adult tissue samples were dissected according to the methods described and prepared in triplicate. The samples used in RT-PCR were from mated males five days after eclosion. The RT-PCR primers ( Table 2 ) were designed using Primer Premier 5.0 (Premier Biosoft, www.premierbiosoft.com ) based on the EST sequences from cDNA library of *B. horsfieldi* . The 18s rRNA was used as the reference gene. PCR experiments were carried out in a PTC- 200 (Bio-Rad, www.bio-rad.com ), and PCR reactions were performed in the following conditions: 94°C for 5 min, 35 cycles of 30 sec at 94°C, 30 sec at 60°C (the melting temperature of the Minus-C OBPs and 18S rRNA), 1 min at 72°C and 72°C for 10 min. The reactions were performed in 25 µL with 300 ng of single-stranded cDNA, 2.0 mM MgCl2, 0.5 mM dNTP, 0.4 µM for each primer, and 1.25 U Taq polymerase (TaKaRa, www.takarabio.com ). PCR products were analyzed by electrophoresis on 1% agarose gel and stained with ethidium bromide to ensure the correct products were being amplified.

**Table 2.**
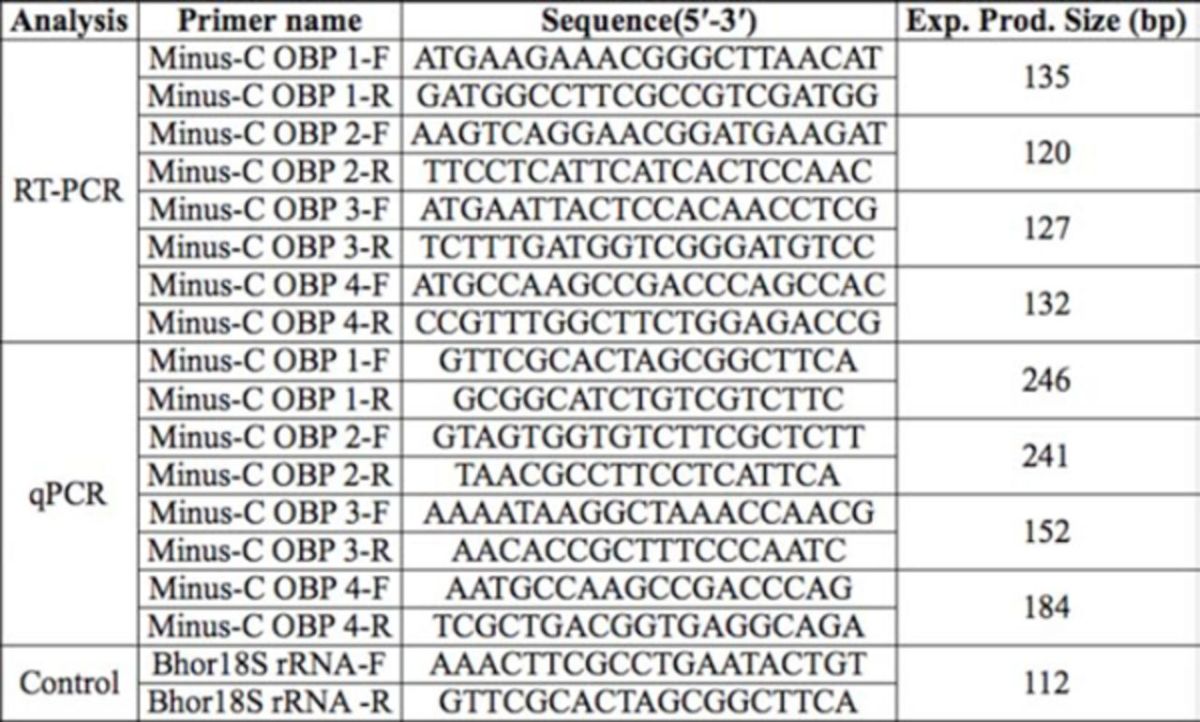
Special primers designed for expression analysis of the OBPs in *Batocera horsfieldi*

Realtime PCR (qPCR) was performed using the ABI 7500 Sequence Detection system (Applied Biosystems). The template cDNA was obtained from unmated or mated males and females of different ages. Specific primer pairs were designed to amplify the Minus-C OBP genes ( Table 2 ). An endogenous control was needed in order to normalize the expression of the target genes and to correct for sample-to-sample variation. Because there are no 18S rRNA and β-actin gene sequences for *B. horsfieldi* in any database, two conserved sequences were selected among the 18S rRNA genes in the Cerambycidae aligned by DNAMAN (Lynnon, www.lynnon.com ) for designing a primer pair for the *B. horsfieldi* 18S rRNA control. The target fragments of Minus-C OBP 1, 2, 3, and 4, and 18S rRNA were expected to be 246 bp, 241 bp, 152 bp, 184 bp, and 112 bp, respectively. The qPCR reaction conditions were 25 µL 2 **×** QuantiTect SYBR Green PCR Master Mix (Qiagen), primer forward, primer reverse, RNasefree water (Millipore, www.millipore.com ), and 500 ng cDNA template per reaction in a final volume of 50 µL. The thermo cycling conditions for qPCR were: 95°C for 3 min, followed by 35 cycles of 30 sec at 94°C, 30 sec at 60°C (the melting temperature of the Minus-C OBPs and 18S rRNA), and 30 sec at 72°C. PCR reactions were performed in triplicate, and the cDNA samples were serially diluted to C0 (1, 101, 102, 103, and 104) times, and the CT values of the Minus-C OBPs and Bhor18S rRNA were measured by qPCR. The data were processed using the relative quantification method. The relative values were measured as 2-∆∆CT (where ∆∆CT = (CTBhor Minus-C OBP – CTBhor18s rRNA) Time x – (CTBhor Minus-C OBP – CTBhor18s rRNA) Time 0) ( Livak and Schmittgen 2001 ).

## Results

### EST sequencing and identification

**Figure 1.**
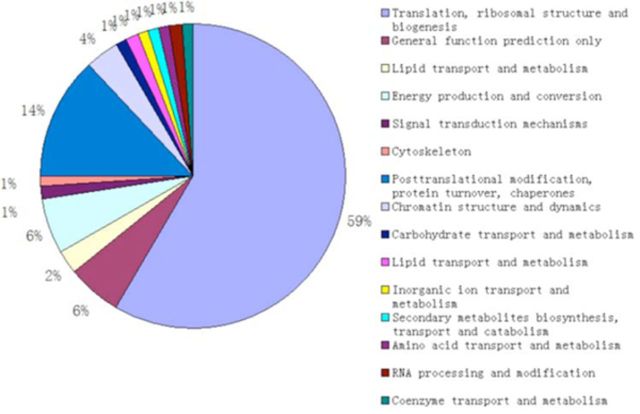
Cluster of orthologous groups of proteins (COG) classification of unigenes in the antennal cDNA library of *Batocera horsfieldi* . High quality figures are available online.

An antennal cDNA library was constructed from a pool of total RNAs extracted from the antennae of male and female *B. horsfieldi* , and the titers of the library was approximately 2.37 × 106 pfu/mL, and the recombinational efficiency was 93.85%. 692 randomly selected clones were sequenced from this antennal cDNA library for further analysis. Of the 649 total clones, 88 clones (the length less than 100 bp) had no obvious ORF. 402 unigenes with an average length of 427 bp were identified. Of the 402 unigenes with an ORF, 106 clones did not produce a significant BLASTx match with a known protein sequence in GenBank. The functional categories of the 402 unigenes are classified and shown in Figure 1 . 58.33% of the unigenes have the function of translation, ribosomal structure, and biogenesis; 13.1% have the function of posttranslational modification, protein turnover, and chaperones; 5.95% have the general function; 5.95% have the function of energy production and conversion; and 3.57% have the function of chromatin structure and dynamics.

### Isolation and characterization of OBP cDNAs from *B. horsfieldi* antennae

**Figure 2.**
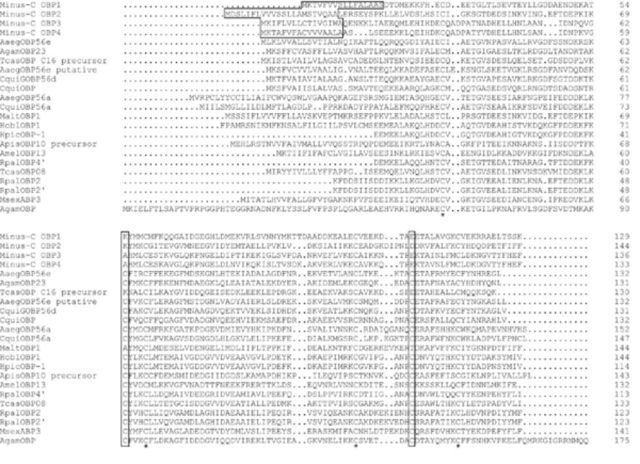
Alignment of predicted amino acid sequences of Minus-C OBPs from the antennal cDNA library with homologous proteins from other insect species (GenBank BLASTP). Predicted signal peptide sequences are boxed; four conserved Cys residues are marked by asterisks; the deletion of the second and the fifth conserved Cys are labeled by enclosing them in rectangles. Sequences used in alignment and accession numbers are Minus-C OBP1 *Batocera horsfieldi* : ADD70030; Minus-C OBP2 *B. horsfieldi* : ADD70031; Minus-C OBP3 *B. horsfieldi* : ADD82416; Minus-C OBP4 *B. horsfieldi* : ADD82417; *Aaeg* OBP56a *Aedes aegypti* : XP_001658810; *Aaeg* OBP56e *A. aegypti* : XP_001655717; *Aaeg* OBP56e putative *A. aegypti* : XP_001655721; *Agam* OBP *Anopheles gambiae* : XP_320225; *Agam* OBP23 *A. gambiae* : XP_320226; *Amel* OBP13 *Apis mellifera* : NP_001035314; *Apis* OBP10 *Acyrthosiphon pisum* : NP_001153525; *Cqui* GOBP56d *Culex quinquefasciatus* : XP_001863135; *Cqui* OBP *C. quinquefasciatus* : XP_001863132; *Cqui* OBP56a *C. quinquefasciatus* : XP_001848933; *Hobl* OBP1 *Holotrichia oblita* : ACX32050; *Hpic* OBP-1 *Heptophylla picea* : BAC07270; *Malt* OBP1 *Monochamus alternatus* : ABR53888; *Msex* ABP3 *Mandu-ca sexta* : AAL60413; *Rpal* OBP2 *Rhynchophorus palmarum* : AAD31875; RpalOBP2' *R. palmarum* : AAD31883; RpalOBP4' *R. palmarum* : AAQ96921; *Tcas* OBP08 *Tribolium castaneum* : EFA04687; *Tcas* OBP C16 precursor *T. castaneum* : NP_001137375. High quality figures are available online.

Following the prediction of their open reading frames and annotation of their biological functions, 68 clones (9.82%) displayed a strong similarity to OBPs or chemosensory proteins (CSPs) from a variety of insect species. After removal of the duplicated sequences, we identified 10 odorant binding proteins and three chemosensory proteins. As for OBPs in insect species, the six-cysteine signature (C1-C6 pattern) is the most striking conservation of amino acids. However, four of the putative OBP genes lacking C2 and C5 ( Figure 2 ) were classified into the Minus-C OBP group, following the naming system proposed by Hekmat-Scafe et al. (2002) . The sequences for the Minus-C OBP1-4 genes have been deposited in GenBank under the following accession numbers: GU575294, GU575295, GU584933, and GU584934. The ORFs of these four Minus-C OBPs were 387 bp, 432 bp, 408 bp, and 399 bp. The computed values of the hydrophobic signal peptides at the N terminus, the theoretical isoelectric point, and molecular mass of the four Minus-C OBPs agreed closely with those obtained for other OBPs ( Table 1 , Figure 2 ).

**Table 1.**
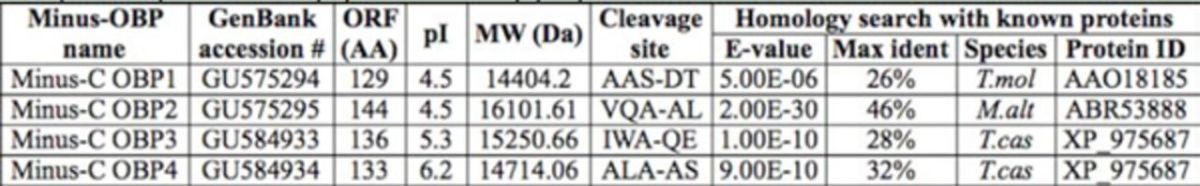
BLAST analysis and prediction of physical chemistry properties of Minus-OBPs.

Note: ORF, open reading frame; pI, isoelectric point; MW, molecular weight; Cleavage site, most likely cleavage site position of signal peptide; E-value, the statistical significance of reported matches; Max ident, the maximum identities of amino acid between Minus-OBP and other insect homologous gene; Species, source species of homologous gene by BLASTX; Protein ID, the accession number of homologs on NCBI; *T.mol* , *Tenebrio molitor* ; *M.alt* , *Monochamus alternates* ; *T.cas, Tribolium castaneum* .

**Figure 3.**
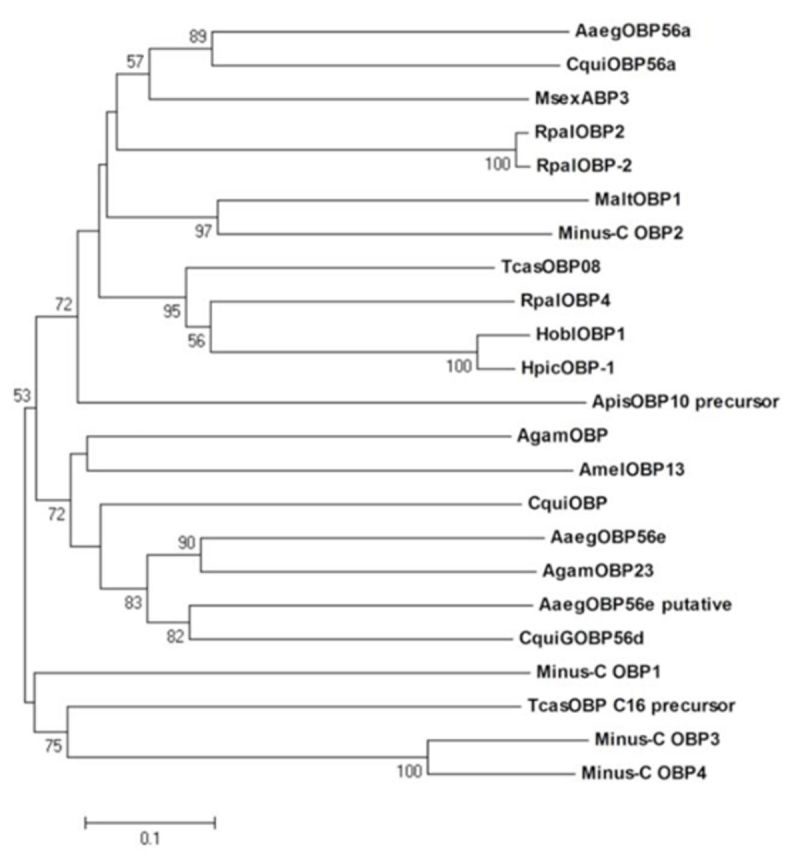
Phylogenetic tree analysis of *Batocera horsfieldi* OBPs (Minus-C OBPs) and other insect species based on amino acid sequence. All OBPs are from the alignment analysis of Figure 2 . The tree was constructed using the neighbor-joining method. Numbers on branches show values of 1500 times replication bootstrap analysis. High quality figures are available online.

We aligned the deduced Minus-C OBP protein sequences with those from *Monochamus alternatus* Hope (Coleoptera: Cerambycidae), *Rhynchophorus palmarum* (L.) (Curculionidae), *Tenebrio molitor* L. (Tenebrionidae), and other insect species. The results showed that the deduced amino acid sequences of OBPs had only four conserved cysteines. Also, we found the amino acids behind the lacking second and fifth cysteine residues were conserved ( Figure 2 ). Amino acid sequence alignments revealed that Minus-OBPs shared 45% identity with each other, and these Minus-C OBPs showed low similarity (28.17%) with OBPs from other insect species. Minus-C OBP1 sequence was homologous to a odorant binding protein of the Coleopteran *T. molitor* (AAO18185), Minus-C OBP2 was homologous to an OBP of *M. alternates* (ABR53888), and Minus-C OBP3 and 4 were homologous to an OBP of *T. castaneum* (XP_975687) ( Table 1 ). In order to better understanding the relationships between these Minus-C OBPs, a phylogenetic tree was constructed using the deduced amino acid sequences ( Figure 3 ). The cladogram indicates that Minus-C OBPs are divided into three separate groups. Minus-C OBP3 and Minus-C OBP4 belonged to a single cluster and were similar to some of the insect OBPs, such as TcasOBP; Minus-C OBP1 belonged to a different cluster and was similar to cluster Minus-C OBP3 and Minus-C OBP4; Minus-C OBP2 belonged to another cluster and was distant from the other three Minus-C OBPs ( Figure 2 ). This result is largely consistent with the alignment above.

### Tissue specificity and spatial expression patterns of Minus-C OBPs

**Figure 4.**
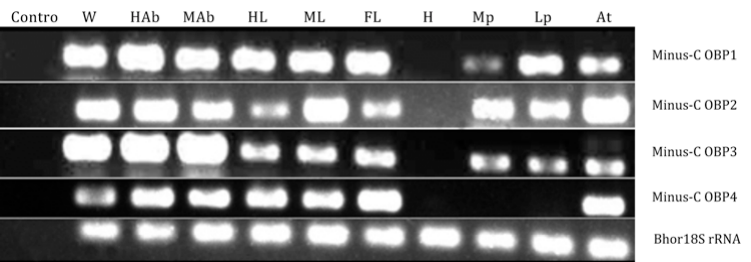
Tissue-specific distribution of Minus-C OBPs. RT-PCRs were performed using cDNAs isolated and synthesized from the different tissues of mated male *Batocera horsfieldi,* five days after eclosion, to examine the expression patterns in the various tissues. Amplification products were analyzed on agarose gel and visualized by UV illumination after staining with ethidium bromide. The PCrproducts were 135 bp in size for Minus-C OBP1, 120 bp for Minus-C OBP2, 127 bp for Minus-C OBP3, 132 bp for Minus-C OBP4, and 112 bp for 18s rRNA (control). Lanes correspond to cDNA or control template derived from: At, antenna; Lp, labial palp; Mp, maxillary palp; H, head (devoid of antennae, labial palps and maxillary palps); FL, foreleg; ML, mid leg; HL, hind leg; MAb, mid abdomen; HAb, hind abdomen; W, wing; control, no template (negative control) to ensure the specificity of the amplification. Amplification of the expressed 18S rRNA cDNA provides an additional positive control for the quality of each cDNA template pool. High quality figures are available online.

Reverse transcription PCR (RT-PCR) experiments were performed using specific primers to determine the distribution of Minus-C OBPs in the various tissues ( Figure 4 ). The integrity of the cDNA templates prepared from different tissues was verified by 18S rRNA gene amplification as a positive control. RT-PCR products of the size predicted for Minus-C OBP1, 2 and 3 were observed in all tested tissues with the exception of the head (without antenna), and RT-PCR products of Minus-C OBP4 were expressed in the antenna, foreleg, middle leg, hind leg, middle abdomen, and hind abdomen, but not in the labial palp, maxillary palp, and head. The data suggested that the genes encoding these proteins are highly expressed in the olfactory tissues (antenna, labial palp, and maxillary palp), as well as in non-olfactory tissues such as the leg, wing, and abdomen. The transcript levels of Minus-OBPs varied between different tissues. Minus-OBP1 had the lowest transcription level in Mp, Minus-OBP2 had the lowest transcription level in the foreleg and hind leg, and Minus-OBP4 had the lowest transcription level in the wing. The transcript levels’ diversity of Minus-OBPs indicate these genes may have various non-olfactory functions.

**Figure 5.**
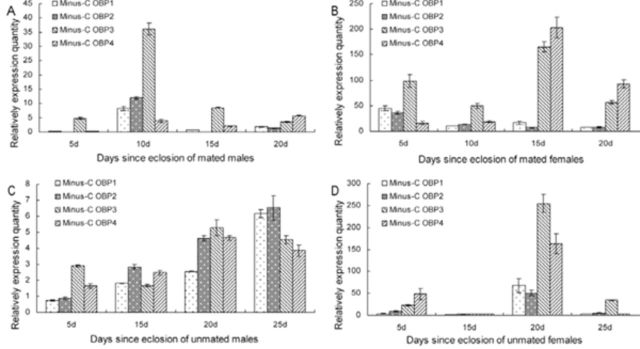
Relative quantity of Minus-C OBPs transcripts expressed from antenna of mated and unmated male and female mature adults by qPCR (mean ± SEM). High quality figures are available online.

Realtime PCR (qPCR) was performed to compare the transcript levels of Minus-C OBPs in antennae of adults from emergence until death. The results show that in all four Minus-OBPs, the transcription level of females was significantly higher than males at the same developmental stages ( Figure 5 ). This was most noticeable in mated females at five days and 15 days after eclosion and unmated females at five days and 20 days, respectively. This result indicated that the genes likely to be involved in olfaction are most highly expressed in the females. This phenomenon can be explained because females look for food and host plants. The expression levels of Minus-C OBP1 and 2 had the lowest transcript levels at five days after eclosion, had the highest level at 10 days, after which they decreased. Minus-C OBP2 had the lowest expression (0.06). Minus-C OBP3 had the highest level at 10 days, reaching 36.08, which was 601 times higher than Minus-C OBP2. The expression levels of Minus- C OBP1 and 2 reached their peak at 25 days, Minus-C OBP3 and 4 reached their peaks at 20 days, and Minus-C OBP2 had the highest expression level (6.52) in unmated males. The transcription levels of Minus-C OBP1 and 2 in mated female antennae had the highest level at five days, then decreased. Minus-C OBP3 and 4 had their highest levels of expression in the 15-day-old mated females, and Minus-C OBP4 had the highest level (203.12) at 15 days, which was 12.62 times higher than that at five days. Unmated females had a higher expression level at five days, then the level decreased and at 15 days they had the lowest level, then at 20 days their highest level was reach, and then levels decreased. Minus-C OBP3 had the highest overall expression level (254.7).

## Discussion

An antennal cDNA library of *B. horsfieldi* was constructed for the first time, and 692 positive clones were sequenced. From approximately 692 clones, we found 296 independent clones that contained ORFs averaging 142 amino acids. All the 296 clones were used to search NCBI with BLASTN and BLASTX. The COG classification shows these clones have different functions: translation, ribosomal structure and biogenesis, posttranslational modification, and protein turnover ( Figure 1 ). As expected, 68 clones (22.97%) shared homologs to OBPs or CSPs from a variety of insect species. After discrimination and classification, we obtained four GOBP genes, two PBP genes, four Minus-C OBP genes, and three CSP genes from the libraries. In addition to an N-terminal signal peptide sequence, these Minus-C OBP genes had only four conserved cysteines, unlike other classical insect OBPs, which have a characteristic pattern of six cysteines. The Minus-C OBP subfamily of *B. horsfieldi* is part of a larger subfamily of insect OBPs that have been reported in some insects, such as *Ceratitis capitata* (Christophides et al. 2000), *T. molitor* ( Graham et al. 2001 ), *Anopheles gambiae* ( Biessmann et al. 2002 ), *Apis mellifera* ( Forêt and Maleszka 2006 ), and *Microplitis mediator* (Zhang et al. 2009). In *Drosophila melanogaster* , the Minus-C subfamily includes seven members; three proteins carry all six conserved cysteines (OBP99a, OBP99d, and OBP83f), and four members of the subfamily (OBP8a, OBP44a, OBP99b, and OBP99c) are missing C2 and C5, indicating that the loss of cysteines C2 and C5 happened after the family diverged from the rest of the insect OBPs (Hekmat- Scafe et al. 2002). The diversity of the OBP gene family suggests that positive selection results in rapid evolutionary changes and functional diversification.

The OBPs of insects have been described as transport proteins that transfer hydrophobic semiochemicals in the sensillum cavity. However, no exact functions of OBPs have yet been clarified, except for the PBPs of moths, where binding studies have confirmed their role in pheromone recognition, specifically their interaction with female pheromones ( Du and Prestwich 1995 ; Biessmann et al. 2002 ). The PBPs also appear to contribute to the excitation of the receptor neurons. Bombykal in combination with the expressed PBP of *Bombyx mori* failed to activate the corresponding receptor neuron of *B. mori* , but did so when combined with one of the PBPs of *A. polyphemus* ( Pophof 2004 ). The OBP LUSH of *D. melanogaster* has been proved to be important in detecting alcohol, knocking out the LUSH gene results in an abnormal attraction to food sources with high concentrations of ethanol, propanol, and butanol ( Kim et al. 1998 ; Kim and Smith 2001 ). In the Minus-C OBP of the moth *Cactoblastis cactorum* , the a10/OS-D has been found expressed in the labial palps (CO2-sensing organs). Some of the *Drosophila* Minus-C OBPs may also have adapted to a non-olfactory function ( Maleszka and Stange 1997 ; Hekmat-Scafe et al. 2002 ), but their exact physiological function still requires further research.

The expression patterns of the Minus-C OBP genes in *B. horsfieldi* may help to characterize the function of these OBPs in future research. The results of the tissue specificity showed that Minus-C OBP1, 2, and 3 were expressed in all dissected tissues except the head (without antennae, labial palps, and maxillary palps), and Minus-C OBP4 was expressed in all tissues tested, except the labial palp, maxillary palp, and head. All Minus-C OBPs had high expression in non-olfactory tissues, such as the legs, wings, and abdomen, but also in olfactory tissues such as the antennae, labial palps, and maxillary palps, except that Minus- C OBP4 was only expressed in the antennae. As for the expression in wings and legs, some papers have reported that Minus-C OBPs are expressed in taste tissues, and these genes may play an important role in taste function and gustatory reorganization. The a10/OS-D, a Minus-C OBP of the moth *C. cactorum* , has been found expressed in the labial palps and can detect the CO2 change in the air ( Maleszka and Stange 1997 ). The distribution diversity of the Minus-OBPs also indicate they may have other functions, not just olfaction.

Realtime PCR was used to evaluate the expression levels and abundance of the identified Minus-C OBPs during various developmental stages and between the sexes. The results showed Minus-C OBPs of adult females expressed at a higher level than that of the males at the same developmental stage, no matter whether they were mated or unmated. The transcription levels of Minus-C OBPs did not change as the male beetle aged over a period of 20–25 days, with the exception of 10-day-old mated males. The data showed mating status had little effect on expression levels of these four genes in male beetles. The age and mating status did affect the expression levels of Minus-C OBPs 3 and 4 in females, with the highest expression levels of these two genes in mated and unmated female occurring on the 15th or 20th day after eclosion, respectively. This phenomenon may be because males look for females, while females look for food and an oviposition site. It has been reported that female insects release a blend of sex pheromones to attract males over long distances, and males detect the released pheromones with extreme sensitivity and selectivity ( Kaissling et al. 1987 ; Baker et al. 2004 ). It also has been reported that age and mating status could affect the expression levels of the PBP gene PxylPBP1 in the diamondback moth, *Plutella xylostella* (Zhang et al. 2009). Other studies in *A.gambiae* and *D. melanogaster* also have shown a correlation between changes in expression of a specific set of genes and behavioral and physiological responses ( Abrantes et al. 2008 ; Zhou et al. 2009 ). However, Merlin et al. (2007) examined expression of one OR and one PBP at nine time points during a 24-hr period post-eclosion in *Spodoptera littoralis* male antennae and found no drastic changes in transcript abundance throughout this period of time. In a study by Soques et al. (2010) , the age and mating status had no effects on the expression levels of two OR genes in the male antenna of *Heliothis virescens* (HvOR13 and HvOR15) and *H. subflexa* (HsOR13 and HsOR15). Taken together, we think that developmental stage and mating status could affect the transcription level of Minus-C OBPs of the beetle, but the effect was different with the different genes.

*B. horsfieldi* adults emerge in the early summer and feed mainly on branches of *R. multiflora* until they are sexually mature. After copulation, the females deposit their eggs at night under the bark of poplar trees before returning to *R. multiflora* to feed again, while the males remain on *R. multiflora* ( Yan et al. 1997 ). Our previous experiments in laboratory olfactometers using beetles deprived of vision have shown that volatiles from *R. multiflora* are attractive to all adults, while volatiles from *Populus* species are only attractive to mated females ( Li et al. 2008 ). In this paper, a cDNA library was successfully constructed from *B. horsfieldi* antennae, and 10 putative OBPs and three CSPs were obtained. These proteins may play an important role in adult female *B. horsfieldi* behavior in looking for food and locating host trees for oviposition. The research on these OBPs may give us a better understanding of the insect olfactory system and possible targets for insect pest control. However, further research on the exact physiological functions and the structural characterization of these OBPs are needed.

## Abbreviations

CSP: chemosensory proteins
EST: expressed sequence tag
OBP: odorant binding protein
OR: odorant receptor
PBP: pheromone binding proteins

## References

[R1] Abrantes P Dimopoulos G Grosso AR Do Rosario VE Silveira H , 2008 . Chloroquine mediated modulation of *Anopheles gambiae* gene expression . PLoS ONE 3 :e 2587 . 10.1371/journal.pone.0002587 PMC2432468 18596975

[R2] Baker TC Ochieng' SA Cossé AA Lee SG Todd JL Quero C Vickers NJ , 2004 . A comparison of responses from olfactory receptor neurons of *Heliothis subflexa* and *Heliothis virescens* to components of their sex pheromone . Journal of Comparative Physiology A: Neuroethology, Sensory, Neural, and Behavioral Physiology 190 : 155- 165. 10.1007/s00359-003-0483-2 14689220

[R3] Biessmann H Walter MF Dimitratos S Woods D . 2002 . Isolation of cDNA clones encoding putative odorant binding proteins from the antenna of the malaria-transmitting mosquito, *Anopheles gambiae* . Insect Molecular Biology 11 : 123 – 132 . 11966877 10.1046/j.1365-2583.2002.00316.x

[R4] Christophides CK Mintzas AC Komitopoulou K . 2000 . Organization evolution and expression of a multigene family encoding putative members of the odorant binding protein family in the medfly *Ceratitis capitata* . Insect Molecular Biology 9 : 185 – 195 . 10762426 10.1046/j.1365-2583.2000.00176.x

[R5] Du G Prestwich GD . 1995 . Protein structure encodes the ligand binding specificity in pheromone binding proteins . Biochemistry 34 : 8726 – 8732 . 7612612 10.1021/bi00027a023

[R6] Forêt S Maleszka R . 2006 . Function and evolution of a gene family encoding odorant binding-like proteins in a social insect, the honey bee ( *Apis mellifera* ) . Genome Research 16 : 1404 – 1413 . 17065610 10.1101/gr.5075706 PMC1626642

[R7] Graham LA Tang W Baust JG Liou Y-C Reid S Davies PL , 2001 . Characterization and cloning of a *Tenebrio molitor* hemolymph protein with sequence similarity to insect odorant-binding proteins . Insect Biochemistry Molecular Biology 31 : 691 – 702 . 11267907 10.1016/s0965-1748(00)00177-6

[R8] Hekmat-Scafe DS Dorit RL Carlson J.R. . 2000 . Molecular evolution of odorant-binding protein genes OS-E and OS-F in *Drosophila* . Genetics 155 : 117 – 127 . 10790388 10.1093/genetics/155.1.117 PMC1461081

[R9] Hekmat-Scafe DS Scafe CR McKinney AJ Tanouye MA . 2002 . Genome-wide analysis of the odorant-binding protein gene family in *Drosophila melanogaster* . Genome Research 12 : 1357 – 1369 . 12213773 10.1101/gr.239402 PMC186648

[R10] Ishida Y Cornel AJ Leal WS . 2002 . Identification and cloning of a female antenna-specific odorant-binding protein in the mosquito *Culex quinquefasciatus* . Journal of Chemical Ecology 28 : 867 – 871 . 12035932 10.1023/a:1015253214273

[R11] Kaissling KE Zack Strausfeld C Rumbo ER . 1987 . Adaptation processes in insect olfactory receptors: Mechanisms and behavioral significance . Annals of the New York Academy of Sciences 510 : 104 – 112 . 3324874 10.1111/j.1749-6632.1987.tb43475.x

[R12] Kim MS Smith DP , 2001 . The invertebrate odorant-binding protein LUSH is required for normal olfactory behavior in *Drosophila* . Chemical Senses 26 : 195 – 199 . 11238251 10.1093/chemse/26.2.195

[R13] Kim MS Repp A Smith DP . 1998 . LUSH odorant-binding protein mediates chemosensory responses to alcohols in *Drosophila melanogaster* . Genetics 150 : 711 – 721 . 9755202 10.1093/genetics/150.2.711 PMC1460366

[R14] Krieger J Mameli M Breer H . 1997 . Elements of the olfactory signaling pathways in insect antenna . Invertebrate Neuroscience 3 : 137 – 144 . 9783439 10.1007/BF02480368

[R15] Li J Wang M-Q Zhang Z-C Chen J Zhang G . 2008 . Behavioral response of *Batocera horsfieldi* adults to plant volatiles . Scientia Silvae Sinicae 44 : 960 – 962 (in Chinese with English summary).

[R16] Livak KJ Schmittgen TD . 2001 . Analysis of relative gene expression data using realtime quantitative PCR and the 2(-Delta Delta C (T)) method . Methods 25 : 402 – 408 . 11846609 10.1006/meth.2001.1262

[R17] Maleszka R Stange G . 1997 . Molecular cloning, by a novel approach, of a cDNA encoding a putative olfactory protein in the labial palps of the moth *Cactoblastis cactorum* . Gene 202 : 39 – 43 . 9427543 10.1016/s0378-1119(97)00448-4

[R18] Merlin C Lucas P Rochat D Francois MC Maibechecoisne M Jacquin-Joly E . 2007 . An antennal circadian clock and circadiam rhythms in peripheral pheromone reception in the moth *Spodoptera littoralis* . Journal of Biological Rhythms 22 : 502 – 514 . 18057325 10.1177/0748730407307737

[R19] Nagnan-Le Meillour P Jacquin-Joly E François MC . 2004 . Identification and molecular cloning of putative odorant-binding proteins from the American palm weevil, *Rhyncophorus palmar* um L . Journal of Chemical Ecology 30 : 1213 – 1223 . 15303324 10.1023/b:joec.0000030273.77407.4d

[R20] Nielsen H Engelbrecht J Brunak S von Heijne G . 1997 . Identification of prokaryotic and eukaryotic signal peptides and prediction of their cleavage sites . Protein Engineering 10 : 1 – 6 . 10.1093/protein/10.1.1 9051728

[R21] Pelosi P Maida R . 1995 . Odorant-binding proteins in insects . Comparative Biochemistry and Physiology, Part B: Biochemistry and Molecular Biology 111 : 503 – 514 . 10.1016/0305-0491(95)00019-5 7613772

[R22] Pelosi P Zhou J-J Ban LP Calvello M . 2006 . Soluble proteins in insect chemical communication . Cellular and Molecular Life Sciences 63 : 1658 – 1676 . 16786224 10.1007/s00018-005-5607-0 PMC11136032

[R23] Pophof B . 2004 . Pheromone-binding proteins contribute to the activation of olfactory receptor neurons in the silkmoths antheraea polyphemus and *Bombyx mori* . Chemical Senses 29 : 117 – 125 . 14977808 10.1093/chemse/bjh012

[R24] Robertson HM Martos R Sears CR Todres EZ Walden KK Nardi JB . 1999 . Diversity of odorant binding proteins revealed by an expressed sequence tag project on male *Manduca sexta* moth antenna . Insect Molecular Biology 8 : 501 – 518 . 10620045 10.1046/j.1365-2583.1999.00146.x

[R25] Soques S Vásquez GM Grozinger CM Gould F . 2010 . Age and mating status do not affect transcript levels of odorant receptor genes in male antenna of *Heliothis virescens* and *Heliothis subflexa* . Journal of Chemical Ecology 36 : 1226 – 1233 . 20890796 10.1007/s10886-010-9863-6

[R26] Steinbrecht RA Laue M Maida R Ziegelberger G . 1996 . Odorant-binding proteins and their role in the detection of plant odours . Entomologia Experimentalis et Applicata 80 : 15 – 18 .

[R27] Tamura K Dudley J Nei M Kumar S . 2007 . MEGA4 : Molecular Evolutionary Genetics Analysis (MEGA) Software Version 4.0 . Molecular Biology and Evolution 24 : 1596 – 1599 . 17488738 10.1093/molbev/msm092

[R28] Thompson JD Higgins DG Gibson TJ . 1994 . CLUSTAL W: improving the sensitivity of progressive multiple sequence alignment through sequence weighting, position-specific gap penalties and weight matrix choice . Nucleic Acids Research 22 : 4673 – 4680 . 7984417 10.1093/nar/22.22.4673 PMC308517

[R29] Vogt RG Riddiford LM . 1981 . Pheromone binding and inactivation by moth antenna . Nature 293 : 161 – 163 . 18074618 10.1038/293161a0

[R30] Vogt RG Lerner MR . 1989 . Two groups of odorant binding proteins in insects suggest specific and general olfactory pathways . Neuroscience 15 : 1290 – 1296 .

[R31] Vogt RG Prestwich GD Lerner MR . 1991 . Odorant-binding protein subfamilies associate with distinct classes of olfactory receptor neurons in insects . Journal of Neurobiology 22 : 74 – 84 . 2010751 10.1002/neu.480220108

[R32] Vogt RG Callahan FE Rogers ME Dickens JC . 1999 . Odorant binding protein diversity and distribution among the insect orders, as indicated by LAP, an OBP-related protein of the true bug *Lygus lineolaris* (Hemiptera, Heteroptera) . Chemical Senses 24 : 481 – 495 . 10576256 10.1093/chemse/24.5.481

[R33] Xu YL He P Zhang L Fang SQ Dong SL Zhang YJ Li F . 2009 . Large-scale identification of odorant-binding proteins and chemosensory proteins from expressed sequence tags in insects . BMC Genomics 10 : 632 . 20034407 10.1186/1471-2164-10-632 PMC2808328

[R34] Xu PX Zwiebel LJ Smith DP . 2003 . Identification of a distinct family of genes encoding atypical odorant-binding proteins in the malaria vector mosquito, *Anopheles gambiae* . Insect Molecular Biology 12 : 549 – 560 . 14986916 10.1046/j.1365-2583.2003.00440.x

[R35] Yan A Ji B Qian J . 1997 . A study on *Batocera horsfieldi* (Hope) . Journal of Nanjing Forestry University 21 : 1 – 6 (in Chinese with English summary).

[R36] Zhang S Zhang YJ Su HH Gao XW Guo YY . 2009a . Identification and expression pattern of putative odorant-binding proteins and chemosensory proteins in antenna of the *Microplitis mediator* (Hymenoptera: Braconidae) . Chemical Senses 34 : 503 – 512 . 19497961 10.1093/chemse/bjp027

[R37] Zhang ZC Wang MQ Zhang G . 2009b . Molecular cloning and expression of pheromone binding protein1 from the diamondback moth, *Plutella xylostella* . Entomologia Experimentalis et Applicata 133 : 136 – 145 .

[R38] Zhou JJ Huang W Zhang G Pickett JA Field LM . 2004 . “Plus-C” odorant-binding protein genes in two *Drosophila* species and the malaria mosquito *Anopheles gambiae* . Gene 327 : 117 – 129 . 14960367 10.1016/j.gene.2003.11.007

[R39] Zhou JJ Vieira FG He XL Smadja C Liu R Rozas J Field LM . 2010 . Genome annotation and comparative analyses of the odorant- binding proteins and chemosensory proteins in the pea aphid *Acyrthosiphon pisum* . Insect Molecular Biology 19(Supplement 2): 113 – 122 . 10.1111/j.1365-2583.2009.00919.x 20482644

[R40] Zhou JJ Zhang GA Huang W Birkett MA Field LM Pickett JA Pelosi P . 2004 . Revisiting the odorant-binding protein LUSH of *Drosophila melanogaster* : evidence for odour recognition and discrimination . FEBS Letters 558 : 23 – 26 . 14759510 10.1016/S0014-5793(03)01521-7

[R41] Zhou S Stone EA Mackay TF Anholt RR . 2009 . Plasticity of the chemoreceptor repertoire in *Drosophila melanogaster* . PLoS Genetics 5 : e 1000681 . 10.1371/journal.pgen.1000681 PMC2750752 19816562

[R42] Zhuge PP Luo SL Wang MQ Zhang G . 2010 . Electrophysiological responses of *Batocera horsfieldi* (Hope) adults to plant volatiles . Journal of Applied Entomology 134 : 600 – 607 .

[R43] Zwiebel LJ Takken W . 2004 . Olfactory regulation of mosquito–host interactions . Insect Biochemistry and Molecular Biology 34 : 645 – 652 . 15242705 10.1016/j.ibmb.2004.03.017 PMC3100215

